# Changes in cardiac-driven perivascular fluid movement around the MCA in a pharmacological model of acute hypertension detected with non-invasive MRI

**DOI:** 10.1177/0271678X231209641

**Published:** 2023-10-24

**Authors:** Phoebe G Evans, Maria Sajic, Yichao Yu, Ian F Harrison, Patrick S Hosford, Ken J Smith, Mark F Lythgoe, Daniel J Stuckey, Jack A Wells

**Affiliations:** 1 UCL Centre for Advanced Biomedical Imaging, Division of Medicine, University College London, London, UK; 2 Department of Neuroinflammation, UCL Queen Square Institute of Neurology, University College London, London, UK; 3 Centre for Cardiovascular and Metabolic Neuroscience, Department of Neuroscience, Physiology and Pharmacology, University College London, London, UK

**Keywords:** CSF, glymphatic, hypertension, MRI, perivascular space

## Abstract

Perivascular spaces mediate a complex interaction between cerebrospinal fluid and brain tissue that may be an important pathway for solute waste clearance. Their structural or functional derangement may contribute to the development of age-related neurogenerative conditions. Here, we employed a non-invasive low *b***-**value diffusion-weighted ECG-gated MRI method to capture perivascular fluid movement around the middle cerebral artery of the anaesthetised rat brain. Using this method, we show that such MRI estimates of perivascular fluid movement directionality are highly sensitive to the cardiac cycle. We then show that these measures of fluid movement directionality are decreased in the angiotensin-II pharmacological model of acute hypertension, with an associated dampening of vessel pulsatility. This translational MRI method may, therefore, be useful to monitor derangement of perivascular fluid movement associated with cardiovascular pathologies, such as hypertension, in order to further our understanding of perivascular function in neurology.

## Introduction

Perivascular spaces (PVS) are fluid-filled channels surrounding selected surface and penetrating vessels and form a key component of the glymphatic hypothesis.^1,2^ The glymphatic system describes a structurally distinct brain-wide fluid transport pathway that facilitates the exchange of cerebrospinal fluid (CSF) with interstitial fluid (ISF) in order to maintain parenchymal homeostasis.^3^ Two-photon microscopy has shown CSF-tracers moving in the direction of blood flow within the PVS around surface pial arteries of the mouse brain.^4^ The CSF-tracers appear to be propelled by the motion of the arterial wall, driven by the cardiac pulse wave.^4^ Impaired arterial wall motion, initiated by angiotensin-II (Ang-II) to induce acute hypertension, provoked a marked reduction in the net flow of CSF-tracers in this region.^4^ These findings highlight the mechanistic relationship between arterial wall motion across the cardiac cycle and perivascular fluid movement at the level of the subarachnoid arteries in the murine brain.

There is currently a lack of non-invasive and clinically translatable techniques to assess fluid movement in the PVS, limiting our understanding of their functional role in disease processes. We previously developed a non-invasive MRI method to assess perivascular fluid movement around the middle cerebral artery (MCA) in the rat brain.^5^ The method applies a long echo time (TE) low *b*-value diffusion-weighted MRI sequence to calculate the pseudo-diffusion coefficient of the perivascular space using multiple ‘motion probing gradient’ directions. As such, this yields a quantitative estimate of perivascular fluid movement, but has mixed sensitivity to, and therefore cannot distinguish, bulk flow from rapid-dispersion or diffusion. Building on this, here, we first capture the influence of cardiac-driven vascular pulsation on measures of perivascular fluid movement using a multi-ECG-delay gated diffusion-weighted MRI sequence (11 timepoints across the cardiac cycle). Based on the marked dependence of measures of PVS fluid directionality on the phase of the cardiac cycle, we then investigate whether we can detect impairment in perivascular fluid movement in an Ang-II pharmacological model of acute hypertension, using non-invasive MRI measurements.

## Methods

### Animal preparation

All animal procedures were approved by UK Home Office, in accordance with the Animals (Scientific Procedures) Act 1986, with data reported in compliance with the ARRIVE guidelines. Adult male Sprague Dawley rats (Charles River Laboratories) were used for all the following experiments: multi-ECG-delay MRI study (n = 10), ultrasound Angiotensin and vehicle control study (n = 9), MRI Ang-II and vehicle control study MRI (n = 12), arterial blood pressure study (n = 1). Temperature and breathing rate were monitored throughout all the experiments using a rectal probe and a respiration pad (SA Instruments). Temperature was maintained at 37 ± 0.5 °C using heated water tubing and warm air flow. Animals weighing 250–350 g were anaesthetised with isoflurane (4% in 0.6 L/min medical air and 0.2 L/min O_2_ for induction; and 2% in 0.4 L/min medical air and 0.2 L/min O2 for maintenance) and placed in a prone position in an MRI cradle with their heads fixed with ear bars, nose cone and bite bar to minimise motion during image acquisition. For ultrasound experiments animals were anaesthetised with isoflurane (4% for induction; 2% for maintenance in 1 L/min oxygen) and positioned in a prone position on a temperature regulated platform with simultaneous ECG recording.

### Magnetic resonance imaging hardware

The multi-ECG study and pharmacological Ang-II induced hypertension MRI experiments were performed using a 9.4 T Varian horizontal bore imaging system (Agilent Inc., Palo Alto, CA) using a 72 mm diameter gradient volume coil for RF transmission and a 4-channel array surface coil receiver (Rapid Biomedical), positioned on top of the head.

#### Non-invasive MRI of perivascular function with multi-ECG delays (n = 10)

**Figure 1.**
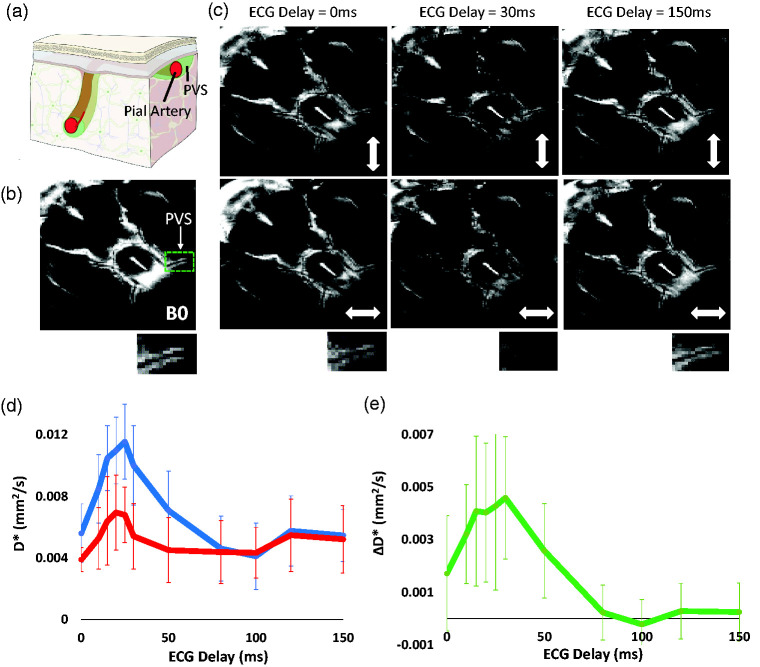
Non-invasive measurement of perivascular fluid movement across the cardiac cycle. (a) Schematic illustration of PVS surrounding a pial artery (reproduced with permission from [https://royalsocietypublishing.org/doi/10.1098/rsif.2019.0572]). (b) Example B0 image at the ventral aspect of the rat brain. Below shows the left PVS around the MCA (highlighted by the green box in b.)) (c) Example ‘Diffusion-weighted’ images at different ECG-delay times with the motion probing gradients applied in orthogonal directions (as indicated by the white arrow). (d) The mean D* across the 10 rats for the parallel (blue) and perpendicular (to the orientation of the PVS - red) alignment of the motion probing gradients across the ECG delays (+/− SD). The data shown was generated by taking the average of the left and right PV space and (e) The mean ΔD* across the 10 rats at increasing ECG delay time (+/− SD). The data shown was generated by taking the average of the left and right PV space.

A 3-plane gradient-echo ‘scout’ image was acquired for subsequent image planning. Parameters for diffusion-weighted MRI sequence: 3 D Fast-Spin-Echo (FSE), FOV 30 mm × 30 mm × 2 mm, TR =4000 ms, echo train length = 32, effective TE = 134 ms, matrix size: 192 × 192 × 2, averages = 4, scan time =3 min 20 seconds. An image was acquired with no diffusion weighting (b0) and then with diffusion gradients (amplitude: 3 G/cm, duration [δ]: 5 ms and separation [Δ]: 26 ms, *b-*value = 43 s/mm^2^) applied in both orthogonal directions i.e., parallel, and perpendicular to the PVS orientation respectively (6 min 40 s scan time). The imaging volume was positioned at the ventral region of the brain using the anatomical reference image to optimally capture the PVS surrounding the MCA (Figure 1(b)). The position and orientation of the imaging slice was manually adjusted to optimally capture the PVS assessed via visual inspection of the scout images. The orientation of the image was adjusted to align the right PVS with the orientation of the frequency encoding (FE) imaging gradients (‘left – right’) and thus the left PVS was approximately aligned to the phase encoding (PE) imaging gradient (‘up – down)’. Thus, when we applied motion probing gradients in the FE, the direction of diffusion weighting was approximately parallel to the right perivascular tract and perpendicular to the left tract; and vice versa when applying diffusion gradients along the PE direction. As a result, the sensitivity for measuring differences in perivascular fluid movement along (i.e. parallel to its main orientation) and perpendicular to both tracts was maximised. The MRI acquisition sequence was triggered at different stages of the cardiac cycle by introducing a range of delays after detection of the R-wave of the ECG signal. Image acquisition was gated to the following delays: 0, 10, 15, 20, 25, 30, 50, 80, 100, 120 and 150 ms. Overall scan time was ∼70 minutes.

*Non-invasive MRI of perivascular fluid movement in a pharmacological model of acute hypertension*

### Pharmacological model of acute hypertension

Angiotensin-II (Sigma-Aldrich) was dissolved in NaCl 0.9% and injected intraperitoneally (i.p.) as a bolus at a volume range 2.25–3 ml to give a dose of 75 ng/kg. Control animals received i.p. injection of NaCl 0.9% saline of equivalent volume.

### Arterial blood pressure measurement

Arterial blood pressure was measured through an arterial catheter in the femoral artery and connected to a physiological monitoring system. Data were sampled at 400 Hz and recorded arterial blood pressure and heart rate simultaneously for a baseline reading across a ten minute period. Ang-II was injected and data were recorded for a further 10-minute period. The average arterial blood pressure was taken for the baseline and post Ang-II.

### Long TE diffusion MRI protocol

The MRI acquisition parameters were similar to that described above but the in-plane image matrix size was lowered to 64 × 64 to provide more signal averaging per unit time in order to maximise sensitivity to changes in perivascular fluid movement that occur in the Ang-II model of hypertension. Pilot experiments yielded highly similar D* estimates in the PVS to that captured using the higher resolution scans described above, owing to the long TE minimising partial volume effects from blood and tissue (as well as the blood ‘flow-through’ effect when using FSE with a multi-echo train). Image acquisition was gated to an ECG delay of 25 ms based on our previous data (see Figure 1) where ΔD* was greatest. The diffusion-weighted imaging parameters were as follows: 3 D Fast-Spin Echo, TR = 4 s, ETL = 32, ESP = 11, effective TE = 134 ms, averages = 8, dummy = 2, FOV = 30 × 30 × 2 mm and scan time 2 min 16 s.

A b0 image was acquired, and two diffusion-weighted images (amplitude: 3 G/cm, duration [δ]: 5 ms and separation [Δ]: 26 ms, b-value = 43 s/mm^2^) and for 7 repetitions (30 minutes scan time). We then delivered Ang-II or saline vehicle (n = 6 each group, groups known to the experimenter, not randomised) via an infusion line whilst the animal was positioned within the magnet bore. The length of the line was long enough to allow manual injection from outside the scanner bore, without having to move the animal bed within the scanner. After delivery of Ang-II, the protocol was repeated (7 repetitions, 30 minutes scan time).

### Image processing and analysis of diffusion weighted MRI data

Based on the b0 images, region of interests (ROI) were manually drawn around the PVS surrounding the left and right branches of the MCA, as well as the subarachnoid space (SAS) and third ventricle. Using the identical ROI, the mean voxel signal was calculated to estimate the b0 signal, and for each read and phase direction. The pseudo-diffusion coefficient (D*) was then calculated for each direction of the applied motion probing gradients using the following equation:

    S = S0exp(−bD∗)

where S is the measured signal at *b* = 43 s/mm2, S0 is the signal taken from the b0 image. The data were averaged across both the left and right perivascular tracts around the MCA when the motion probing gradient was applied parallel and perpendicular to their main orientation respectively to generate an overall measurement of D* parallel and perpendicular to the central orientation of the PVS across both regions.

The ΔD* was calculated by taking the difference between the D* at parallel and perpendicular directions as an estimate of the directionality of perivascular fluid movement (analogous to the difference between axial and radial diffusivity in diffusion tensor imaging [DTI]). For the pharmacological study we calculated the average ΔD* in the PVS at baseline and after Ang-II injection for each individual animal as a summary measure of the directionality of perivascular fluid movement at baseline and following Ang-II/vehicle.

### Ultrasound

Ultrasound was used to estimate changes in large arterial vessel pulsatillity following Ang-II at the level of the carotid artery. Ultrasound imaging was performed using a Vevo 2100 system (VisualSonics, California, CA, USA) with a MS550D 30‐MHz transducer. The B-mode trace located the left and right common carotid artery (CCA) vessels in axial orientation. M-mode and doppler recordings were made with the ultrasound transducer perpendicular (rotated 90°) to the CCA to image in a longitudinal axis near the bifurcation. M-mode was used to measure arterial wall motion by taking the difference in CCA vessel diameter at systole and diastole phases. Data were acquired at 5-minute intervals over a 20-minute baseline. In addition, the time interval between peak blood velocity and the R-wave in the ECG signal was recorded to gauge possible changes in pulse wave velocity. After the baseline, Ang-II (n = 6) or saline vehicle control (n = 3) was administered intraperitoneally (i.p.) and both wall motion and the time interval between peak blood flow velocity and the R-wave were acquired for at 5-minute intervals for 20 minutes. The average of wall motion and timing measures were taken at baseline and then post Ang-II.

### Statistical analysis

Measurements are reported as mean +/− SD or the individual subject data are shown. Statistical tests were performed using the non-parametric sign-rank paired test (1-tailed) to investigate: i) for the multi-ECG delay study, differences in the mean D* between the parallel and perpendicular orientation of the motion probing gradients (averaged across all ECG delays in both the parallel and perpendicular directions); ii) for the pharmacological study, decreases in wall motion after Ang-II and decreases in ΔD* after Ang-II or vehicle. Here, non-parametric tests were used because the n-numbers made it difficult to test for normality with any certainty. All statistical analyses were performed on Matlab (2022 b).

## Results

### Non-invasive MRI of perivascular function with multi-ECG delays

An imaging volume was positioned to capture the perivascular space surrounding the middle cerebral artery at the ventral aspect of the rat brain (Figure 1(a) and (b)). At selected ECG delay times, diffusion-weighted images show markedly greater signal attenuation in the PVS when motion probing gradients were applied in the direction parallel to their main orientation compared to perpendicular, reflecting the known principal direction of fluid movement within the PVS (Figure 1(c)). Overall, the mean D* across all the ECG delays was significantly greater in the parallel vs perpendicular direction (p < 0.01). We measured a peak in D* in parallel and perpendicular directions at ∼25 ms after the R-wave in the cardiac cycle (Figure 1(d)). This observation likely reflects arterial pulsation during the systole phase of the cardiac cycle, accounting for the time taken for the pulse wave to travel from the heart to the MCA. In turn, ΔD* (Figure 1(e)), a measure of the directionality of perivascular fluid movement, was found to exhibit striking sensitivity to the ECG delay time, ranging from ∼0.004 mms^−2^ during systole to ∼0 during diastole. Other fluid compartments such as subarachnoid CSF and the 3rd ventricle show clear dependence on the cardiac cycle but with no dependence on the direction of the motion probing gradients, as expected (supplementary Figure 1). Based on the data shown in Figure 1(e), we surmise, therefore, that ΔD* represents a sensitive measure of perivascular fluid movement directionality driven by vascular pulsation across the cardiac cycle. Thus, we hypothesised that measures of ΔD* will be reduced where there is impaired vascular wall motion, as previously observed in a rodent model of Ang-II-induced acute hypertension using direct, invasive, measures.^4^

### Non-invasive MRI of perivascular fluid movement in a pharmacological model of acute hypertension

**Figure 2.**
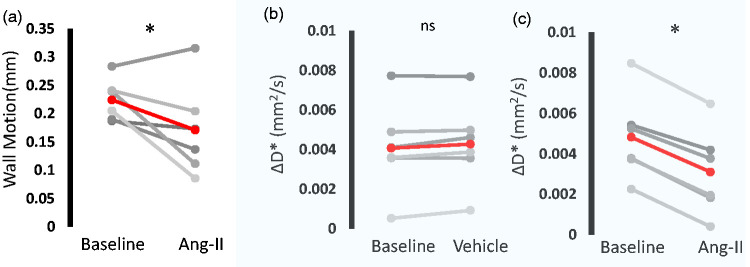
Pharmacological hypertension reduces directionality of PV fluid movement measured with non-invasive MRI. a) Estimated wall motion of the carotid artery across the cardiac cycle before and after Ang-II (each grey line represents an individual rat, group average in red). Mean ΔD* over the baseline period and b) vehicle and c) Ang-II (each grey line represents an individual rat, group average in red). *p<0.05 sign-rank, 1-tailed.

Following infusion of Ang-II, mean arterial blood pressure was increased by 45% (mean arterial blood pressure: 88 mmHg to 128 mmHg - supplementary Figure 2). Ultrasound of the carotid artery was used to gauge changes to cardiac pulsatility in the large arteries (as the MRI measure of PVS fluid movement is taken at the MCA) following Ang-II administration. Heart rate was 364 bpm at baseline and remained relatively similar after Ang-II, recorded at 353 bpm. The average wall motion at baseline was 0.22 ± 0.02 mm with the mean across the cohort decreasing to 0.17 ± 0.03 mm (p < 0.05) after Ang-II (Figure 2(a)). The time interval between peak blood velocity and the R-wave in the ECG signal did not change between baseline and after Ang-II and the average was measured at 40.64 ms and 40.36 ms (supplementary Figure 3) respectively, suggesting that pulse wave velocity is not impacted relative to the timescale of the MRI measurements taken over the cardiac cycle (Figure 1(d)). In contrast, no changes in arterial wall motion were recorded after saline vehicle (supplementary Figure 4). Non-invasive MRI measurements of PVS fluid movement directionality (ΔD*) were taken before and after Ang-II or vehicle at the peak of cardiac driven PVS fluid movement (25 ms – based on Figure 1d). There was no change in ΔD* before and after vehicle bolus (0.004 and 0.004 mm^2^/s, p > 0.05) (Figure 2(b)). ΔD* was significantly reduced after Ang-II (0.005 and 0.003 mm^2^/s, p < 0.05), a decrease that was highly consistent across the cohort (Figure 2(c)).

## Discussion

In this study we take non-invasive measures of perivascular fluid movement around the MCA of an anaesthetised rat model using a long-TE, low *b* ***-*** value diffusion-MRI sequence. Measures of fluid directionality (ΔD*) across the cardiac cycle demonstrate marked dependence on cardiac phase, with ΔD* ranging from ∼0.004 mm^2^/sec during systole to ∼0 (ie no measured fluid directionality) during diastole. This suggests that this approach can capture the action of cardiac-driven vascular pulsation to propel perivascular fluid movement and by extension, that this measure will be sensitive to reduced pulsatility. In turn, we then demonstrate sensitivity of the method to detect perivascular derangement associated with impaired vascular wall motion in an Ang-II model of acute hypertension. This work demonstrates that we can now detect changes in perivascular fluid movement associated with reduced vascular pulsatility using non-invasive MRI.

Prior studies have interrogated CSF movement in the PVS surrounding a pial artery in the mouse brain using two-photon microscopy and particle tracking of fluorescent microspheres.^4^ They observed that the microspheres moved in a forward direction along the PVS following pulsations due to systole, with little backward motion during diastole. These findings provide evidence that cardiac-driven motion of the vessel wall propels CSF flow in the direction of blood flow, around the surface arteries. Following questions regarding the influence of contrast agent injection on the observed tracer kinetics, more recent studies have addressed this methodological concern by simultaneous injection and removal of the equivalent amount of fluid to maintain constant ICP. They found that CSF tracer motion were highly consistent with previous observations, with no measurable increase in ICP or CSF volume.^6^ We have previously shown that the MRI technique employed here is sensitive to perivascular function during the action of arterial pulsation,^5^ and here we extend this to assess perivascular fluid movement across the whole cardiac cycle. The D* measurement showed a peak at 20–30 ms delay after the R-wave which we surmise to reflect the time for the pulse wave generated from each heartbeat to propagate through the circulatory system and reach the brain’s blood vessels.^4^ Thus, here, we demonstrate cardiac pulsation-coupled perivascular fluid movement modulated over the cardiac cycle, measured non-invasively as was recently reported in the human brain using this translational measurement.^7^

Based on previous observations,^6^ we assumed that the principal direction of fluid movement in the PVS runs parallel to the orientation of the blood vessel. Therefore, we manually positioned two motion-probing gradients to be aligned parallel and perpendicular to the vessel to estimate perivascular fluid movement parallel and perpendicular to the main orientation of the MCA, respectively. The difference in perivascular D* between the two diffusion-weighted directions (ΔD*) during arterial pulsation (20–30 ms) demonstrates a dominant movement of fluid alongside the orientation of the MCA, as expected. At longer delays (100–150 ms) which corresponded to diastole, we recorded negligible difference in the directionality of PV fluid movement (i.e., mean ΔD* ∼0). Interestingly, the dynamic changes in ΔD* across the cardiac cycle (Figure 1(e)) show close resemblance to direct measures of PV flow using two photon imaging (see, for example, Figure 3(b) in Mestre et al.,^4^ in the mouse brain). Thus, based on these data (Figure 1(e)), we propose that ECG-gated measures of ΔD* may represent a sensitive, non-invasive surrogate measure of ‘perivascular pumping’ efficacy at the level of the subarachnoid arteries. We next investigated possible changes in ΔD* in a model of acute transient hypertension, adapted from a mouse model in which robust decreases in PV flow were recorded, attributed to a concomitant reduction in wall motion pulsatility.^4^ We measured a consistent decrease in ΔD* after Ang-II-induced hypertension, where relative blood pressure increase was comparable to human hypertension defined as >110–115 mmHg,^8^ with no change recorded after vehicle (Figure 2). To our knowledge, this is the first time that an impairment of perivascular function associated with hypertension has been captured using non-invasive measurements.

Here, we are measuring perivascular fluid movement surrounding a large pial vessel at the base of the brain as an ‘upstream’ perivascular region. Several studies have shown that this region is a key entry route of CSF-tracers towards the brain and that contrast-agent based measures of CSF movement at this site correlate with more downstream markers of CSF-tracer delivery to the tissue under a range of conditions.^9,10^ Thus, albeit a large surface vessel with surrounding PVS, this region represents a promising surrogate marker of CSF influx into the tissue. Here, we opted for two motion probing gradients in order to maximise sensitivity to changes in perivascular fluid movement in the parallel and perpendicular PVS orientation due to cardiac phase (Figure 1) or hypertension (Figure 2) (ie greater no of repetitions vs more motion probing gradient orientations). Future studies, however, may wish to use >=6 motion probing gradient directions in order to generate a tensor, so that the measurements are not dependent on the manual positioning of the motion probing gradients relative to the orientation of the PVS/vessel. Such an approach may also enable similar measures across the whole brain rather than a single region.

As described in our initial depiction of the method,^5^ measures of D* represent a surrogate, quantitate, marker of CSF movement, non-specific to flow coherence (i.e., bulk flow vs pulsatile driven fluid dispersion with no net-flow vs diffusion). Nonetheless, recent studies have further explored the source of D* contrast in the CSF and shown a strong correlation between D* from diffusion-weighted signals and CSF peak velocity using Monte Carlo simulations.^11^ Therefore measurements of D* may serve as a quantitative marker of fluid movement in the PVS and have the potential to be a useful non-invasive biomarker for derangement of PV function linked to pathological states of the brain.

It is important to consider the following limitations of the present work: i) US measures of changes in vessel pulsatility following Ang-II were taken not at the MCA (where the MRI measures of perivascular fluid movement take place), but at the carotid artery in the neck (due to the difficulty of imaging the MCA with US because of the limited penetration through the skull). Nonetheless, decreases in the pulsatilty recorded at the carotid confirmed the effect of Ang-II to decrease vessel pulsatility in the large arteries in the anaesthetised rat model used here (as was recently reported in subarachnoid arteries in an Ang-II mouse model^9^) ii) MR measures of perivascular fluid movement were taken at a single ECG-delay time to maximise sensitivity to changes after Ang-II or vehicle. The ECG delay time was chosen at 25 ms based on the data presented in Figure 1 where a peak in ΔD* was detected at ∼25 ms. Importantly, however, no changes in the pulse wave velocity before or after Ang-II were detected (estimated at the carotid arteries from the time interval between peak blood velocity and the R-wave in the ECG signal), suggesting that this remained a reliable measure of ‘peak’ ΔD* across the cardiac cycle after Ang-II. In this work Ang-II was given as an IP bolus. This produced a transient increase with a mean BP of 88 mmHg at baseline to an average of 128 mmHg in the 10 minutes post-bolus injection. Although the peak increase is likely to be a relatively moderate compared to what could be achieved with an IV injection, the increased BP is nonetheless relevant to the changes that are observed in clinical hypertension.

As shown in both Figure 1(d) and Figure 2(b)/(c), we record a fairly large range of baseline D* values across the different subjects. Whether this variance reflects genuine biological variation or whether this is primarily methodological in origin remains unknown at this time. Interestingly, the degree of between-animal variance in baseline D* values appears similar to the degree of between-animal variance in measures of perivascular velocity via direct assessment using two-photon imaging in the mouse brain [Mestre et al., 2018]. However, our experience with the method leads us to believe that the manual positioning of the imaging slice and the orientation of the motion probing gradients provide an important source of methodological variance in D* measures. Nonetheless, our pilot data (supplementary Figure 5) indicated good temporal stability of D* measurements providing reassurance that the method would be sensitive to drug driven changes in the same imaging session as evidenced by the data presented in Figure 2 and thus this study was appropriately designed to test the proposed hypotheses. Future studies that aim to compare different cohorts may, however, wish to further understand these possible sources of measurement variability to improve baseline D* measurement precision.

To date, a lack of non-invasive imaging techniques has limited our understanding of perivascular function in the human brain and their precise role in brain-clearance and disease pathogenesis. In the human brain, PVSs become significantly more visible on MRI when they are structurally enlarged, indicating perivascular dysfunction and possible impairment of brain clearance via the proposed glymphatic system.^12^ The PVS enlarges with ageing and vascular risk factors such as hypertension. The non-invasive nature of this technique could allow better understanding of perivascular dysfunction in the human brain to disentangle the mechanisms driving early-stage pathogenesis of SVD. Promisingly, recent studies have demonstrated the translational capability of this technique where brain-wide measures of cardiac cycle influenced CSF-movement in the PVS were captured.^7^ Moreover, Bito et al., demonstrated the reproducibility and feasibility of low b-value diffusion tensor imaging for measurement of local pseudo-random flow of CSF in the human brain.^13^ This method could improve our understanding of CSF physiology via exploring changes in local perivascular CSF flow dynamics, working towards novel therapeutic strategies to stimulate brain clearance processes.

In conclusion, here we show that a long-TE low *b-*value diffusion weighted MRI approach can capture measures of perivascular fluid movement across the cardiac cycle around the MCA in the anaesthetised rat brain. In a pharmacologically induced model of hypertension, we detected a marked reduction in measures of perivascular fluid movement directionality. Our results highlight the capability of non-invasive MRI to detect and quantify functional changes to perivascular fluid movement, as opposed to structural deformations. This technique has the potential to be utilised to assess early changes in perivascular fluid movement to better understand the mechanisms that underly age-related neurodegenerative disease.

## Supplemental Material

sj-pdf-1-jcb-10.1177_0271678X231209641 - Supplemental material for Changes in cardiac-driven perivascular fluid movement around the MCA in a pharmacological model of acute hypertension detected with non-invasive MRI Supplemental material, sj-pdf-1-jcb-10.1177_0271678X231209641 for Changes in cardiac-driven perivascular fluid movement around the MCA in a pharmacological model of acute hypertension detected with non-invasive MRI by Phoebe G Evans, Maria Sajic, Yichao Yu, Ian F Harrison, Patrick S Hosford, Ken J Smith, Mark F Lythgoe, Daniel J Stuckey and Jack A Wells: on behalf of the CONTRAST consortium in Journal of Cerebral Blood Flow & Metabolism

sj-pdf-2-jcb-10.1177_0271678X231209641 - Supplemental material for Changes in cardiac-driven perivascular fluid movement around the MCA in a pharmacological model of acute hypertension detected with non-invasive MRI Supplemental material, sj-pdf-2-jcb-10.1177_0271678X231209641 for Changes in cardiac-driven perivascular fluid movement around the MCA in a pharmacological model of acute hypertension detected with non-invasive MRI by Phoebe G Evans, Maria Sajic, Yichao Yu, Ian F Harrison, Patrick S Hosford, Ken J Smith, Mark F Lythgoe, Daniel J Stuckey and Jack A Wells: on behalf of the CONTRAST consortium in Journal of Cerebral Blood Flow & Metabolism

sj-pdf-3-jcb-10.1177_0271678X231209641 - Supplemental material for Changes in cardiac-driven perivascular fluid movement around the MCA in a pharmacological model of acute hypertension detected with non-invasive MRI Supplemental material, sj-pdf-3-jcb-10.1177_0271678X231209641 for Changes in cardiac-driven perivascular fluid movement around the MCA in a pharmacological model of acute hypertension detected with non-invasive MRI by Phoebe G Evans, Maria Sajic, Yichao Yu, Ian F Harrison, Patrick S Hosford, Ken J Smith, Mark F Lythgoe, Daniel J Stuckey and Jack A Wells: on behalf of the CONTRAST consortium in Journal of Cerebral Blood Flow & Metabolism

sj-pdf-4-jcb-10.1177_0271678X231209641 - Supplemental material for Changes in cardiac-driven perivascular fluid movement around the MCA in a pharmacological model of acute hypertension detected with non-invasive MRI Supplemental material, sj-pdf-4-jcb-10.1177_0271678X231209641 for Changes in cardiac-driven perivascular fluid movement around the MCA in a pharmacological model of acute hypertension detected with non-invasive MRI by Phoebe G Evans, Maria Sajic, Yichao Yu, Ian F Harrison, Patrick S Hosford, Ken J Smith, Mark F Lythgoe, Daniel J Stuckey and Jack A Wells: on behalf of the CONTRAST consortium in Journal of Cerebral Blood Flow & Metabolism

sj-pdf-5-jcb-10.1177_0271678X231209641 - Supplemental material for Changes in cardiac-driven perivascular fluid movement around the MCA in a pharmacological model of acute hypertension detected with non-invasive MRI Supplemental material, sj-pdf-5-jcb-10.1177_0271678X231209641 for Changes in cardiac-driven perivascular fluid movement around the MCA in a pharmacological model of acute hypertension detected with non-invasive MRI by Phoebe G Evans, Maria Sajic, Yichao Yu, Ian F Harrison, Patrick S Hosford, Ken J Smith, Mark F Lythgoe, Daniel J Stuckey and Jack A Wells: on behalf of the CONTRAST consortium in Journal of Cerebral Blood Flow & Metabolism
