# Supplementary figures and images for: Changes in cardiac-driven perivascular fluid movement around the MCA in a pharmacological model of acute hypertension detected with non-invasive MRI
Source: J Cereb Blood Flow Metab. 2023 Oct 24;44(4):508–15. doi: 10.1177/0271678X231209641 (PMC10981406; doi:10.1177/0271678X231209641)

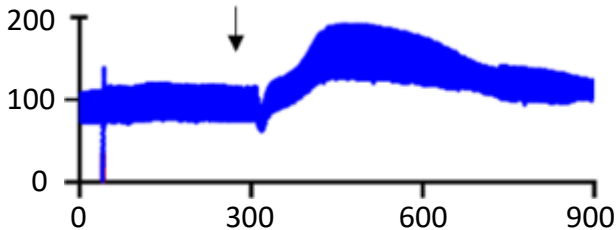

**Supplementary Figure 2.** Blood pressure trace following delivery of Ang-II (arrow).

Supplement: sj-pdf-2-jcb-10.1177_0271678X231209641 - Supplemental material for Changes in cardiac-driven perivascular fluid movement around the MCA in a pharmacological model of acute hypertension detected with non-invasive MRI [file sj-pdf-2-jcb-10.1177_0271678X231209641.pdf]
