# Supplementary material for: Changes in cardiac-driven perivascular fluid movement around the MCA in a pharmacological model of acute hypertension detected with non-invasive MRI
Source: J Cereb Blood Flow Metab. 2023 Oct 24;44(4):508–15. doi: 10.1177/0271678X231209641 (PMC10981406; doi:10.1177/0271678X231209641)
Supplement: sj-pdf-5-jcb-10.1177_0271678X231209641 - Supplemental material for Changes in cardiac-driven perivascular fluid movement around the MCA in a pharmacological model of acute hypertension detected with non-invasive MRI [file sj-pdf-5-jcb-10.1177_0271678X231209641.pdf]

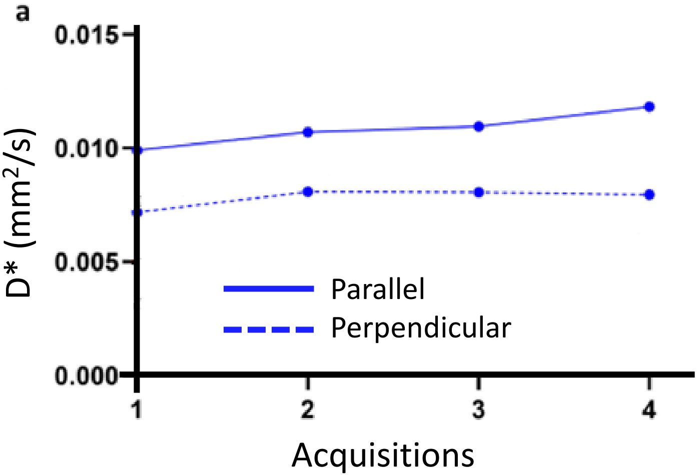

Supplementary Figure 5.

Estimated wall motion of carotid artery before and after saline vehicle (25ms ECG delay time)
